# Hyaluronan of Different Molecular Weights Exerts Distinct Therapeutic Effects on Bleomycin-Induced Acute Respiratory Distress Syndrome

**DOI:** 10.3390/ijms27020580

**Published:** 2026-01-06

**Authors:** Shu-Ting Peng, Chia-Yu Lai, Tsui-Ling Ko, Chun-Hsiang Hsu, I-Yuan Chen, You-Cheng Jiang, Kuo-An Chu, Yu-Show Fu

**Affiliations:** 1 Department of Anatomy and Cell Biology, School of Medicine, National Yang Ming Chiao Tung University, Taipei 11221, Taiwan; stpeng65565@nycu.edu.tw; 2 Institute of Anatomy and Cell Biology, School of Medicine, National Yang Ming Chiao Tung University, Taipei 11221, Taiwan; lilianart29@gmail.com; 3 School of Medicine, College of Medicine, National Sun Yat-sen University, Kaohsiung 804, Taiwan; kate819b@g-mail.nsysu.edu.tw; 4 Division of Chest Medicine, Department of Internal Medicine, Kaohsiung Veterans General Hospital, Kaohsiung 813414, Taiwan; chhsu1642@vghks.gov.tw (C.-H.H.); elyc9295@gmail.com (I.-Y.C.); goodduck.jiang@gmail.com (Y.-C.J.); 5 School of Nursing, Fooyin University, Kaohsiung 831301, Taiwan; 6 Department of Nursing, Shu-Zen Junior College of Medicine and Management, Kaohsiung 821, Taiwan

**Keywords:** acute respiratory distress syndrome, acute lung injury, hyaluronan, lung function, macrophage, immune storm

## Abstract

Acute respiratory distress syndrome (ARDS) is a fatal inflammatory lung disorder with few effective treatments. Hyaluronan (HA), a major extracellular matrix component, exhibits diverse biological activities depending on its molecular weight. This study aimed to evaluate the therapeutic potential of HA of various molecular weights in a rat model of ARDS. ARDS was induced in rats via the intratracheal instillation of 5 mg of bleomycin. Seven days later, when ARDS symptoms developed, low (LHA), medium (MHA), high (HHA), and mixed (MIX HA) hyaluronan were intratracheally administered seven times from Days 7 to 28. On Day 7, arterial oxygen saturation (SpO_2_) and the partial pressure of oxygen (PaO_2_) decreased, carbon dioxide levels increased, the respiratory rate increased, and extensive lung cell infiltration was observed, confirming successful ARDS induction. LHA and MIX HA improved the SpO_2_ and PaO_2_, and the latter increased lung and alveolar volume, reduced infiltration, and normalized breathing. All HA types attenuated collagen deposition and M1 macrophage activity, while MIX HA enhanced M2 polarization and upregulated MMP-2, MMP-9, and TLR-4. LHA increased VEGF and EGF expression. These findings demonstrate that different-weight HAs provide partial ARDS protection via distinct mechanisms. MIX HA shows synergistic effects, restoring and improving lung structure and function, respectively, representing a promising ARDS therapy.

## 1. Introduction

Acute respiratory distress syndrome (ARDS) is a severe and life-threatening manifestation of acute lung injury, characterized by diffuse pulmonary inflammation, hypoxemia, and respiratory failure. Despite advances in intensive care and the use of corticosteroids, current treatments remain largely supportive in nature, mortality rates are high, and survivors often suffer from pulmonary fibrosis and impaired quality of life. Thus, ARDS continues to represent a major challenge in critical care medicine. Several animal models of lung injury have been developed, including radiation exposure [1], silica inhalation [2], asbestos exposure [3], bacterial infection [4], intratracheal administration of bacterial lipopolysaccharide (LPS) [5], and drug-induced injury such as bleomycin (BLM) administration [6,7]. However, most models do not fully reproduce the clinical features of ARDS as defined by the Berlin criteria [8,9]. A recently modified intrabronchial BLM model produced an animal model of severe, reproducible lung injury with hypoxemia, inflammatory storm, cell infiltration, and epithelial loss, thereby providing a more reliable platform for evaluating novel ARDS therapies [10].

Our previous studies demonstrated that intratracheal transplantation of human umbilical cord-derived mesenchymal stromal cells (HUMSCs) could attenuate pulmonary fibrosis through three mechanisms: suppressing inflammation and myofibroblast activation, promoting macrophage-derived MMP-9 to degrade collagen, and upregulating TLR-4 to stimulate epithelial regeneration [11,12,13]. These findings suggest that HUMSC-secreted factors may play key roles in lung repair and raise the possibility that HUMSCs could also be effective for treating ARDS.

Wharton’s jelly, the native niche of HUMSCs, is one of the richest sources of hyaluronic acid (HA), which is synthesized and secreted by HUMSCs [11,14,15,16]. Therefore, we hypothesized that HA contributes to the therapeutic mechanisms of HUMSCs. HA is classified into four major categories based on molecular weight: high-molecular-weight HA (HMW-HA, MW ≥ 10^3^ kDa), medium-molecular-weight HA (MMW-HA, MW 250–1000 kDa), low-molecular-weight HA (LMW-HA, MW 10–250 kDa), and HA oligomers (<10 kDa). Interestingly, different-molecular-weight HA exerts distinct physiological and biological functions [17]. In this study, we tested different-molecular-weight HAs using a severe BLM-induced ARDS rat model to evaluate their therapeutic efficacy.

## 2. Results

### 2.1. Administration of LHA and MIX HA Increased Arterial SpO_2_ Levels in Rats with Severe ARDS

**Figure 1:**
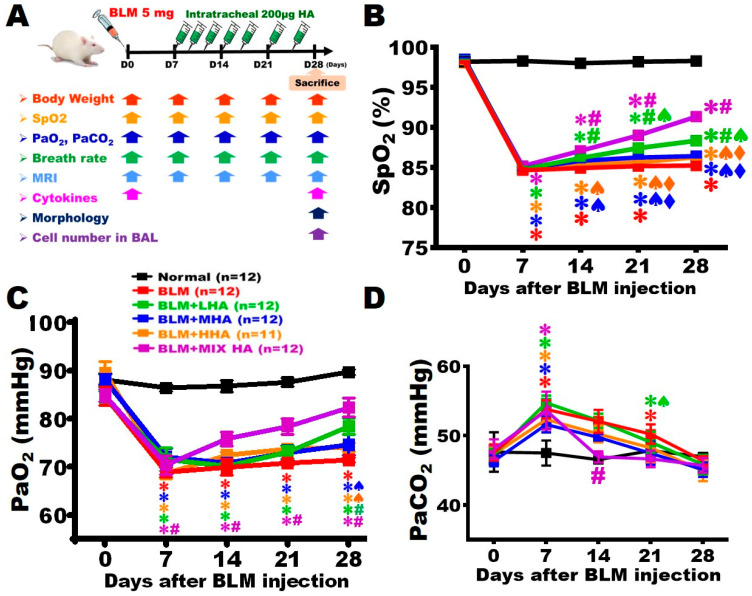
Administration of LHA and MIX HA elevated arterial SpO_2_ in rats with severe ARDS. (**A**) The study flowchart and experimental groups are shown. BLM was injected into the rats’ left bronchus on Day 0, and the animals were sacrificed on Day 28. (**B**) Alterations of arterial SpO_2_ of each group were quantified at different time points. The detection of arterial PaO_2_ (**C**) and PaCO_2_ (**D**) by drawing arterial blood from each group to assess lung function. (*n* = 11 for BLM + HHA group and *n* = 12 for other groups). ✱ vs. the Normal group at the same time, *p* < 0.05; **#** vs. the BLM group at the same time, *p* < 0.05; ♠ vs. the BLM + MIX HA group at the same time, *p* < 0.05; ⧫ vs. the BLM + LHA group at the same time, *p* < 0.05.

The arterial SpO_2_ was assessed using a pulse oximeter to evaluate the gas exchange function of the lung. The results showed that SpO_2_ levels were maintained at around 98.2% throughout the 28-day study period in the Normal group (Figure 1B). On Day 7 post-BLM damage, the SpO_2_ level significantly decreased to 84.3 ± 0.7% in all other study groups. The level of arterial SpO_2_ of the BLM group did not improve until Day 28, at which point it was markedly lower than that of the Normal group (Figure 1A,B). The arterial SpO_2_ levels of the BLM + MHA and BLM + HHA groups were about 86% on Day 28, which were not statistically different from those of the BLM group. The BLM + LHA and BLM + MIX HA groups displayed significant increases in arterial SpO_2_ between Day 14 and Day 28 when compared with the BLM group. Notably, the arterial SpO_2_ level of the BLM + MIX HA was also significantly higher than that of the BLM + LHA group on Days 21 and 28 (Figure 1A,B).

In addition, the arterial PaO_2_ level was between 86.4 and 89.7 mmHg before BLM damage (Day 0) (Figure 1C). On Day 7 post-BLM damage, each study group showed substantial reductions in arterial PaO_2_ level to between 68.9 and 72.0 mmHg. The arterial PaO_2_ level of the BLM group remained unchanged until Day 28 and was significantly lower than that in the Normal group at this point (Figure 1C). The arterial PaO_2_ level was 74.6 ± 4.4 mmHg in the BLM + MHA group and 74.1 ± 1.9 mmHg in the BLM + HHA group; although slight increases were observed, these values did not significantly differ from those of the BLM group. The arterial PaO_2_ level of the BLM + LHA group increased to 78.4 ± 4.0 mmHg on Day 28, significantly higher than that of the BLM group at this point. In the BLM + MIX HA group, the arterial PaO_2_ level was substantially increased on Day 14 compared with that in the BLM group, and this trend of improvement was sustained until Day 28 (Figure 1C).

On Day 0 (before BLM injury), the arterial PaCO_2_ level was between 46.4 and 47.9 mmHg (Figure 1D). The arterial PaCO_2_ levels of all study groups were significantly higher than those of the Normal group on Day 7 post-BLM damage. On Day 14, the arterial PaCO_2_ level remained elevated in all study groups except for the BLM + MIX HA group. The arterial PaCO_2_ level of the BLM + MIX HA group significantly decreased compared to that of the BLM group and was close to that of the Normal group. The remaining study groups (BLM, BLM + LHA, BLM + MHA, and BLM + HHA) showed no statistical differences in the level of arterial PaCO_2_ compared with that of the Normal group on Day 21 (Figure 1D).

### 2.2. Administration of LHA and MIX HA Alleviated the Respiratory Rate in Rats with Severe ARDS

**Figure 2:**
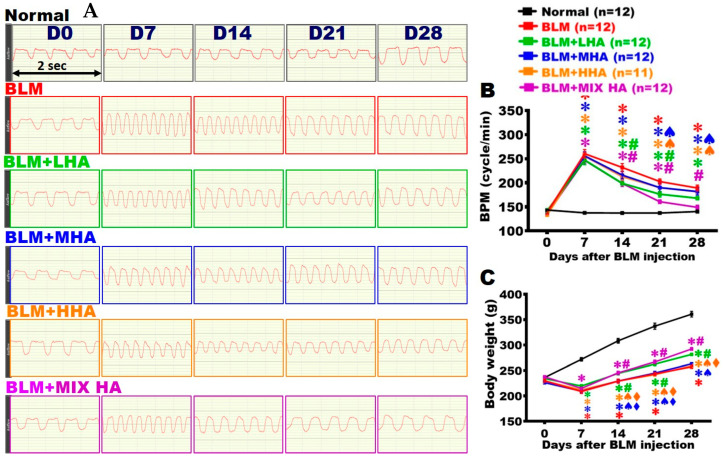
Administration of LHA and MIX HA alleviated respiratory rate in rats with severe ARDS. (**A**) The lung function assessment was to record the number of breaths per minute at different time points. The times of breaths within 2 s for each study group are shown. (**B**) Quantitative analysis of respiratory rates in each study group. (**C**) The body weight of each group was quantified at different time points. (*n* = 11 for BLM + HHA group and *n* = 12 for other groups). ✱ vs. the Normal group at the same time, *p* < 0.05; **#** vs. the BLM group at the same time, *p* < 0.05; ♠ vs. the BLM + MIX HA group at the same time, *p* < 0.05; ⧫ vs. the BLM + LHA group at the same time, *p* < 0.05.

Breaths per minute (BPM) were counted to estimate lung function. We found that the Normal group maintained a stable respiratory rate from Day 0 to Day 28, namely around 4–5 times every 2 s (Figure 2A). On Day 7 post-BLM damage, the respiratory rates in all study groups except for the Normal group had markedly increased. From Day 7 to Day 28, the respiratory rate in the BLM group significantly increased compared to that in the Normal group (Figure 2A,B). For the BLM + MHA and BLM + HHA groups, the respiratory rates from Day 7 to Day 28 were similar to those of the BLM group. The BLM + LHA group’s respiratory rate had significantly decreased on Day 14 compared to that of the BLM group; however, its rate was still higher than that of the Normal group. In the BLM + MIX HA group, the respiratory rate significantly decreased since Day 14 compared with that of the BLM group; this trend continued until Day 28, and no statistical differences were found on Day 28 compared to that of the Normal group (Figure 2A,B).

### 2.3. Administration of LHA and MIX HA Increased the Body Weights of Rats with Severe ARDS

Body weight measurement can serve as an indicator of overall health status and systemic inflammatory conditions. The body weights of rats in the Normal group increased over time. For the other study groups, the rats’ body weights stopped increasing from Day 0 to Day 7 post-BLM damage. Although body weights slightly increased over time after Day 7, they were significantly lower than those of the Normal group, and this trend continued until Day 28. From Day 14 to Day 28, the body weights of rats in the BLM + LHA and BLM + MIX HA groups statistically increased compared with those of rats in the BLM group (Figure 2C).

### 2.4. MRI Revealed That the Administration of LHA and MIX HA Increased Air Space in Rats with Severe ARDS

**Figure 3:**
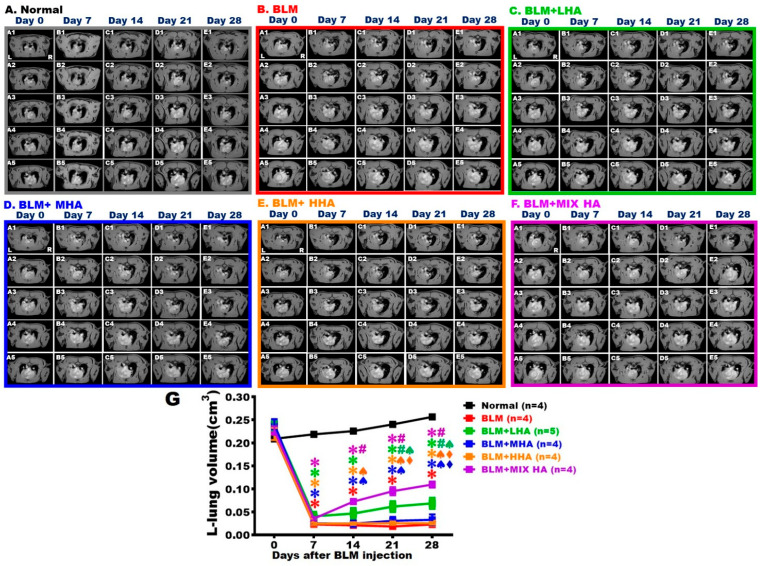
MRI revealed that administration of LHA and MIX HA increased air space in rats with severe ARDS. The MRI of rats is shown, including the Normal group (**A**), the BLM + LHA group (**B**), the BLM + LHA group (**C**), the BLM + MHA group (**D**), the BLM + HHA group (**E**), and the BLM + MIX HA group (**F**). Five MRI scan images from each group were taken at different time points. L represents the left lung and R represents the right lung. The carina was set as a landmark for image positioning. In addition to the slice containing the carina, four more images after the carina were collected. These five images were summed for the quantification of the black alveolar space to represent the left lung alveolar volume of the rat (**G**). (*n* = 5 for BLM + LHA group and *n* = 4 for other groups). ✱ vs. the Normal group at the same time, *p* < 0.05; **#** vs. the BLM group at the same time, *p* < 0.05; ♠ vs. the BLM + MIX HA group at the same time, *p* < 0.05; ⧫ vs. the BLM + LHA group at the same time, *p* < 0.05.

Five MRI images were obtained for each rat to represent and quantify changes in left lung alveolar volume at various time points. The black area represented the location where alveoli were located, and the white area indicated the consolidated tissues. The results showed that the left thoracic cavity in the Normal group was continuously dominated by the black space of the alveoli (Figure 3A,G). In the BLM group, the alveolar space in the left lung was significantly reduced on Day 7 due to inflammatory responses and cellular infiltration, which appeared as white signals on the images; therefore, the alveolar volume was markedly decreased. This phenomenon persisted until Day 28. (Figure 3B,G) For the BLM + MHA and BLM + HHA groups, air space in the left lung remained similar to that of the BLM group from Day 7 to Day 28; the values were not statistically different from those of the BLM group but were significantly lower than those in the Normal group (Figure 3D,E,G). On Day 7 post-damage, white signals appeared in the air space of the left lung in the BLM + LHA and BLM + MIX HA groups. Compared to the BLM group, air space in the left lung started to increase on Day 14 for the BLM + MIX HA group and Day 21 for the BLM + LHA group; these increases continued until Day 28. Moreover, air space in the BLM + MIX HA group was significantly higher than that in the BLM + LHA group on Day 28 (Figure 3C,F,G).

### 2.5. Overall Appearance and H&E Staining Revealed That the Administration of HA Mitigated Left Lung Shrinkage in Rats with Severe ARDS

**Figure 4:**
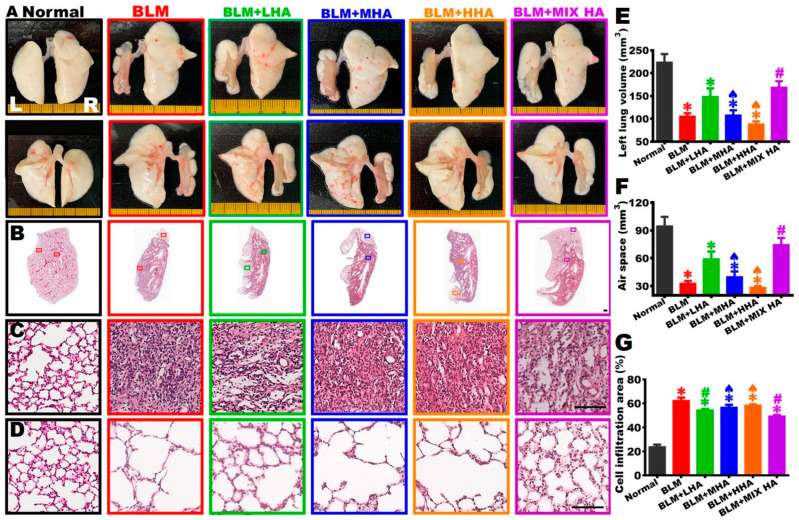
Administration of MIX HA improved the lung volume in rats with severe ARDS. (**A**) The ventral (**upper panel**) and dorsal (**lower panel**) images of the left and right lungs of each group were taken on Day 28. (**B**) The left lung tissue sections were stained with H&E and are shown at low magnification. The images at high magnification showed the central area (**C**) and peripheral area (**D**) of the left lung. The total volume of the left lung was quantified by summing data from all left lung sections. The results showed that, in the BLM + MIX HA group, the left lung volume increased (**E**), the air space increased (**F**), and the cell infiltration area decreased (**G**). (*n* = 4 for Normal group and *n* = 5 for other groups). ✱ vs. the Normal group at the same time, *p* < 0.05; **#** vs. the BLM group at the same time, *p* < 0.05; ♠ vs. the BLM + MIX HA group at the same time, *p* < 0.05.

H&E staining demonstrated pathological alterations in lung tissue. On Day 28, rats were sacrificed and perfused to obtain left and right lungs to observe the overall appearance of the ventral and dorsal sides. The photographs showed that white alveolar structures with intact and smooth appearances were observed in both the left and right lungs of rats in the Normal group. The left lung markedly shrank in the BLM group, with scarce alveolar structure present in the outer region and no alveoli, but pathological tissues were present in the central region of the left lung. Only the left lung of rats in the BLM + MIX HA group appeared to have a relatively larger alveolar area (Figure 4A).

The left lung tissues of each group were subjected to serial sections and H&E staining. According to the images taken at low (Figure 4B) and high (central area: Figure 4C; peripheral region: Figure 4D) magnifications, the results show that the volume of the left lung was larger, the ratio of alveolar space was higher, and connective tissues only appeared near the bronchus but rarely between alveoli in the Normal group. In all other study groups, substantial cell infiltration was observed in the central area, and alveoli were only observed in the outer region of the left lung (Figure 4C,D). Furthermore, by summing data from all left lung sections stained with H&E, statistical analyses revealed that the total volume of the left lung significantly shrank in the BLM group, and the volume of the alveolar structure decreased substantially, with consolidated tissues occupying 62% of the total left lung volume (Figure 4E–G). In the BLM + MHA and BLM + HHA groups, the total volume of the left lung, alveolar space, and percentages of the cell-infiltrating region were not significantly different from those of the BLM group. The total volume of the left lung in the BLM + MIX HA group significantly increased; the volume of air space was higher and close to that of the Normal group. The volumes of connective tissues and the cell infiltration area in the BLM + MIX HA group significantly decreased compared with those of the BLM group, suggesting that administering HA at mixed molecular weights could alleviate cell infiltration, increase overall left lung volume, and restore the alveolar space of the left lung (Figure 4E–G).

### 2.6. Administration of HA Reduced Collagen Deposition in Rats with Severe ARDS

**Figure 5:**
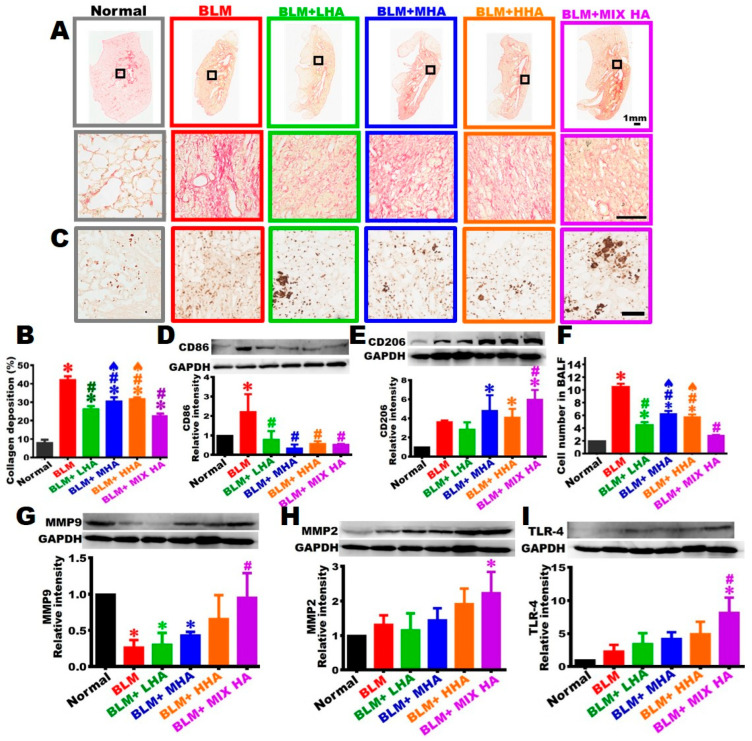
Underlying mechanisms of HA in the treatment of severe ARDS in rats. (**A**) The left lung tissue sections of each group were subjected to Sirius red staining. The area stained with Sirius red represents the location of collagen. The **upper panel** shows images at low magnification, and the **lower panel** shows images at high magnification. (**B**) Quantification of the area occupied by collagen in the left lung. (**C**) The left lung sections of each group were obtained on Day 28 for immunohistochemical staining using anti-ED1 antibody to label macrophages. Western blot analysis was performed using an anti-CD86 antibody to identify M1 macrophages (**D**), and an anti-CD206 antibody to identify M2 macrophages (**E**) in the left lung. (**F**) On Day 28, the cell number in BALF obtained from each group was quantified. Western blotting was performed to quantify the levels of MMP-9 (**G**), MMP-2 (**H**), and TLR-4 (**I**) in the left lungs. (*n* = 3 for all groups). ✱ vs. the Normal group at the same time, *p* < 0.05; **#** vs. the BLM group at the same time, *p* < 0.05; ♠ vs. the BLM + MIX HA group at the same time, *p* < 0.05.

The left lung sections of each group were subjected to Sirius red staining to label collagen red. The images were shown from low to high magnifications, with red indicating where collagen was located (Figure 5A). In the left lung of the Normal group, collagen mainly appeared around the bronchus and vessels. In the left lung of the BLM group, the red area representing collagen significantly increased in size, indicating increased collagen deposition in the lung (Figure 5A,C). When HA was administered to rats in the BLM + LHA, BLM + MHA, BLM + HHA, and BLM + MIX HA groups, collagen deposition in the left lung markedly decreased compared to that of the BLM group; however, collagen deposition was still significantly increased compared to that in the Normal group (Figure 5A,C).

### 2.7. Administration of MIX HA Promoted the Activation of M2 Macrophages to Perform Anti-Inflammatory Responses in Rats with Severe ARDS

The left lung sections of each group were subjected to immunohistochemical staining with anti-ED1 antibody to label macrophages. The results indicated that only a limited number of macrophages with small morphology existed in the left lung of the Normal group. In the left lung tissues of the BLM group, a large number of small macrophages appeared. In the left lung tissues of the BLM + LHA, BLM + MHA, BLM + HHA, and BLM + MIX HA groups, a number of macrophages with relatively small morphology remained; however, some macrophages with large morphology were observed, especially in the left lungs of the BLM + LHA and BLM + MIX HA groups, where large macrophages were scattered in the connective tissues and between alveoli. At a high magnification, these large macrophages contained apparent cytoplasmic granules (Figure 5B).

Western blotting was performed using the anti-CD86 antibody to label pro-inflammatory M1 macrophages. The results revealed that CD86 levels increased in the left lung of the BLM group. Nevertheless, the expression level of left lung CD86 in the BLM + LHA, BLM + MHA, BLM + HHA, and BLM + MIX HA groups statistically differed from that of the BLM group (Figure 5D), suggesting that administering HA at various molecular weights could reduce the differentiation and transformation of M1 macrophages in the left lung of rats with severe ARDS.

Furthermore, Western blotting using an anti-CD206 antibody was conducted to label anti-inflammatory M2 macrophages. The content of CD206 in the left lung of the BLM group slightly increased but did not significantly differ from that of the Normal group. The levels of left lung CD206 in the BLM + MHA, BLM + HHA, and BLM + MIX HA groups were statistically higher than that of the Normal group; among the groups, the CD206 level of the BLM + MIX HA group was significantly higher than that of the BLM group (Figure 5E), suggesting that medium-, high-, or mixed-molecular-weight HA could promote the differentiation and transformation of M2 macrophages to perform anti-inflammatory functions in the left lung of rats with severe ARDS.

### 2.8. The Comparison of Cell Number in Bronchoalveolar Lavage

The cell number measured in BALF was used to estimate the amplitude of pulmonary inflammation. On Day 28, left lung BALF was collected from each study group for cell counting. The results showed that cell counts significantly increased in left lung BALF in the BLM group, whereas cell counts for the BLM + LHA, BLM + MHA, and BLM + HHA groups significantly decreased compared to those of the BLM group but significantly increased compared to those of the Normal group. Additionally, cell counts in the BALF of the BLM + MHA and BLM + HHA groups were higher than those of the BLM + MIX HA group. Cell counts in the BALF of the BLM + MIX HA group significantly decreased compared to those of the BLM group and did not statistically differ from those of the Normal group. Therefore, the results suggest that administering HA could alleviate inflammatory responses in the lungs of severe ARDS rats, which would explain why the cell counts in the left lung decreased (Figure 5F).

### 2.9. Administration of HA Stimulated Matrix Metallopeptidase 9 (MMP-9) Synthesis to Reduce Inflammatory Responses and Promote Collagen Degradation in the Left Lung of Rats with Severe ARDS

The MMP-9 and MMP-2 contents in the left lung of each study group were quantified to evaluate their involvement in collagen degradation. The results showed that left lung MMP-9 levels in the BLM, BLM + LHA, and BLM + MHA groups significantly decreased compared with those of the Normal group. The MMP-9 expression level in the left lung of the BLM + MIX HA group was significantly higher than that in the BLM group; however, this merely represented a return to the level observed in the Normal group (Figure 5G). Additionally, the results for MMP-2 showed that the MMP-2 level in the left lung of the BLM group did not significantly differ from that of the Normal group, whereas the MMP-2 level in the left lung of the BLM + MIX HA group significantly increased compared with that of the Normal group (Figure 5H).

### 2.10. Administration of HA Altered TLR-4 Expression and Accelerated Alveolar Epithelial Cell Regeneration in the Left Lungs of Rats with Severe ARDS

TLR4 expression was quantified to assess pulmonary inflammation or alveolar epithelial regeneration. Western blotting with an anti-TLR-4 antibody was performed to assess the TLR-4 expression of the left lungs for each study group. Only a small amount of TLR-4 was expressed in the left lungs of the Normal and BLM groups. For the BLM + LHA, BLM + MHA, and BLM + HHA groups, although the TLR-4 level slightly increased in the left lung, no statistical difference was observed compared with the Normal and BLM groups. However, the TLR-4 level in the BLM + MIX HA group substantially increased and was different from those in the Normal and BLM groups to a statistically significant extent (Figure 5I). These results suggest that HA could promote TLR-4 biogenesis and accelerate the regeneration and restoration of alveolar epithelial cells in the left lung of rats with severe ARDS.

### 2.11. Administration of HA Changed Immune Reactions in Rats with Severe ARDS

**Figure 6:**
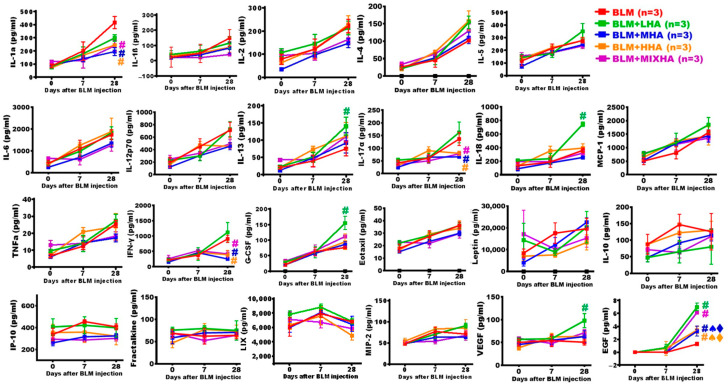
Administration of HA changed inflammatory reactions in rats with severe ARDS. The changes in serum cytokines at different time points indicated that some pro-inflammatory factors increased rapidly at Day 7 following BLM damage, and this increasing trend persisted until Day 28. (*n* = 3 for all groups). **#** vs. the BLM group at the same time, *p* < 0.05; ♠ vs. the BLM + MIX HA group at the same time, *p* < 0.05; ⧫ vs. the BLM + LHA group at the same time, *p* < 0.05.

To investigate changes in pro-inflammatory and anti-inflammatory cytokines, the serum samples of each study group were obtained at various time points for cytokine analyses. The results revealed that the levels of several pro-inflammatory cytokines and chemokines markedly increased at Day 7 after BLM damage, including IL-1α, IL-1β, IL-2, IL-4, IL-5, IL-6, IL-12p70, IL-13, IL-17α, MCP-1, TNFα, IFN-γ, G-CSF, Eotaxin, and Leptin. Moreover, the levels of these pro-inflammatory mediators continued to progressively increase up to Day 28. However, the expression levels of IL-10, IL-18, IP-10, Fractalkine, LIX, MIP-2, VEGF, and EGF showed no significant changes before or after injury. On Day 28, the levels of IFN-γ, IL-1α and IL-17α in the sera of the BLM + MHA, BLM + HHA, and BLM + MIX HA groups were significantly lower than those in the BLM group. The levels of IL-13, IL-18, G-CSF, VEGF, and EGF in the BLM + LHA group were significantly higher compared with those of the BLM group. EGF expression in the BLM + MIX HA group was comparable to that in the BLM + LHA group and was higher than that in the other groups. In addition, EGF expression in the BLM + MHA and BLM + HHA groups was also higher than that in the BLM group. These findings indicate that administration of HA is sufficient to enhance EGF expression (Figure 6).

## 3. Discussion

The intratracheal instillation of BLM into the left bronchus induced ARDS in rats. Starting on Day 7 post-injury, different molecular weights of hyaluronan (HA) were administered intratracheally for seven treatments, and the animals were sacrificed on Day 28. The results demonstrated that ARDS rats treated with MIX HA exhibited the most pronounced improvements in pulmonary function and lung morphology, followed by those treated with LHA. In the BLM + MIX HA group, the expression levels of anti-inflammatory effect, MMPs, and TLR-4 in lung tissue markedly increased; however, in the BLM + LHA group, the expression levels of IL-13, IL-18, G-CSF, VEGF, and EGF significantly increased.

To achieve a reproducible and consistent effect on pathological change and to ensure a better quality of life and higher survival rate for rats with ARDS, BLM (5 mg/rat) was injected into each rat’s left bronchus. The rat was then rotated toward its left side by 60 degrees to establish a severe left lung ARDS animal model for precisely evaluating the effect of medication or stem cell therapy. The animal model with ARDS was employed in this study; on Day 7 following injury, the rat’s arterial SpO_2_ level decreased to around 83%, the arterial PaO_2_ level fell to 69 mmHg, the respiratory rate increased to 310 cycles/min, substantial cell infiltration appeared among alveolar tissues, alveoli disappeared, and an immune storm was triggered. These signs resembled the clinical characteristics defined in [10]. According to the Berlin criteria, the PaO_2_/FiO_2_ ratio should be equal to or lower than 200 mmHg. Since providing a long-term oxygen supply using an oxygen mask is difficult in rats, only the results of arterial PaO_2_ were shown in this study. Only the measurements of SpO_2_ and arterial blood gas analysis for PaO_2_ were performed under inhalation anesthesia (isoflurane), whereas respiratory rate measurements were conducted without anesthesia. The use of anesthesia allowed rats in all groups to remain calm and resulted in a reduced heart rate. However, in the Normal group, SpO_2_ values measured under anesthesia were comparable to those obtained without anesthesia (98–99%), with no significant differences observed. Therefore, short-term inhalation anesthesia did not markedly affect the assessment of blood oxygenation.

In previous studies involving animal models of lung injury, hyaluronan (HA) has been administered either via intratracheal instillation (0.2–0.3% in saline) or aerosol inhalation (0.1% in saline). Regardless of the molecular weight of HA (2000 kDa, 1500–1700 kDa, 800–1200 kDa, 300–500 kDa, or 150 kDa), these studies consistently demonstrated effective attenuation of inflammation and amelioration of lung injury [18,19,20,21,22,23,24,25,26]. Notably, the molecular weights of HA used in these studies are comparable to the low-, medium-, and high-molecular-weight HA applied in the present study. Therefore, the MIX HA group in this study was formulated using an equal ratio (1:1:1) of low-, medium-, and high-molecular-weight HA. Nevertheless, it would be worthwhile for future studies to further investigate whether different HA ratios or dosages produce distinct therapeutic effects. Furthermore, most previous studies administered HA at the time of lung injury, resulting in significant therapeutic effects. In the present study, HA treatment was initiated on Day 7 after lung injury, when severe lung damage and reduced blood oxygenation were already evident. Under these conditions, HA administration was still able to attenuate lung injury. We suggest that earlier administration of HA, such as at the time of injury or within the first three days, may further enhance its therapeutic efficacy. This will be an important topic for future investigation.

We previously transplanted human umbilical mesenchymal stem cells (HUMSCs) and reversed pulmonary fibrosis in rats. Three mechanisms were involved in HUMSC-mediated treatment: reducing inflammatory reactions and inhibiting myofibroblast activation, promoting extracellular matrix degradation, and stimulating alveolar epithelial cell regeneration [11]. In fact, these mechanisms may also participate in ARDS treatment. It has been reported that the umbilical cord contains a high amount of HA [11,14,16]; hence, it was speculated that HA might play a crucial role in treating pulmonary fibrosis and ARDS. Interestingly, the mechanisms involved in mixed HA’s ability to treat ARDS were sometimes different from those involved in treating pulmonary fibrosis through HUMSC transplantation. In our previous study, HUMSCs transplantation promoted MMP-9 expression and activity on Day 21 following BLM damage, but no significant alterations were seen for MMP-2 [11]. Nevertheless, in this study, administering MIX HA significantly increased both MMP-9 and MMP-2 levels in the lung on Day 28, suggesting that MMP-2 may participate in the early restoration of lung damage, while MMP-9 may be involved in the middle-to-late stage of recovery.

Additionally, MMP-9 affects macrophage polarization and plays a critical role in inflammatory responses in the lung. Cabrera et al. intratracheally injected BLM into transgenic mice in which macrophages highly expressed MMP-9. The results showed that cell infiltration by neutrophils and lymphocytes eased and the severity of pulmonary fibrosis decreased, suggesting that macrophages expressing MMP-9 have anti-inflammation and anti-fibrosis capabilities [27]. Our previous study also revealed that the transplantation of HUMSCs increased MMP-9 biogenesis in lung tissues and that MMP-9 predominantly existed in M2 macrophages with larger morphological sizes and larger cytoplasmic granules [11]. The results of this study also showed that MMP-9 and MMP-2 levels in the lungs of rats in the BLM + MIX HA group were higher than those in the Normal or BLM groups. Most past studies reported that high-molecular-weight HA had anti-inflammatory effects and displayed abilities for restoring acute lung damage [24,28,29], while low-molecular-weight HA could trigger M1 macrophage formation and promote inflammatory responses during the pathogenesis of cancer [17]. Similar conclusions were reached by Zhang et al., as the binding of medium-molecular-weight HA and TLR-4 promoted macrophages to differentiate into the M2-like phenotype [30]. Our results showed that administering MIX HA promoted the expression of M2 macrophages. Moreover, treatment with LHA, MHA, HHA, and MIX HA reduced M1 activation (as shown via anti-CD86 Western blotting) and significantly reduced the number of inflammatory cells in the BALF, possibly due to the relatively higher molecular weight of LHA in this study.

TLR-4 plays an important role in the regeneration and restoration of alveolar epithelial cells [31,32]. We previously discovered that the transplantation of HUMSCs markedly increased TLR-4 levels in the lung, which were highly expressed around alveoli [11]. In this study, although administering MHA and HHA slightly increased the expression levels of MMP9, MMP2, and TLR-4, these changes did not achieve statistical significance. It was, therefore, hypothesized that the combined administration of MHA and HHA might synergistically enhance their expression. Indeed, treatment with MIX HA significantly upregulated MMP9, MMP2, and TLR-4 levels.

Acute respiratory distress syndrome (ARDS) triggers a systemic immune storm characterized by excessive activation of pro-inflammatory cytokines and inflammatory mediators, leading to downstream alveolar injury. Previous studies have explored therapeutic strategies for modulating and suppressing these pro-inflammatory cytokines to mitigate ARDS progression [33,34]. In a PM2.5-induced rat model of lung inflammation, high-molecular-weight HA (HMW-HA, 1500–1700 kDa) enhanced anti-inflammatory responses and inhibited alveolar epithelial apoptosis [26]. In a clinical trial, HMW-HA also reduced systemic cytokine levels and improved respiratory failure after acute lung injury [21], indicating its anti-inflammatory role during the acute phase of injury. Our cytokine assay results demonstrated that ARDS rats developed a severe cytokine storm on Days 7 and 28 after lung injury. On Day 28, treatment with MHA, HHA, and MIX HA reduced IFN-γ, IL-1α and IL-17α expression, thereby attenuating the subsequent amplification of inflammation. In contrast, administering LHA markedly increased the expression levels of IL-18, G-CSF, VEGF, and EGF. Treatment with G-CSF prevented LPS-induced acute lung injury by modulating the balance between pro- and anti-inflammatory cytokines [35]. In previous studies, the absence of VEGF signaling had detrimental effects on fetal lung development, and its neutralization was shown to impair lung maturation, reduce surfactant production, and cause both capillary and alveolar hypoplasia in animal models [36]. Conversely, the upregulation of VEGF expression increased lung angiogenesis and promoted alveolar growth in hyperoxia-induced injury in rat lungs [37]. Moreover, VEGF enhanced compensatory lung growth through upregulating EGF [38]. Regarding EGF, its administration attenuated lung inflammation and injury, probably through activating EGFR, leading to the suppression of NF-κB signaling, promotion of cell proliferation, and inhibition of apoptosis [39], as well as the prevention of systemic inflammatory response syndrome [40]. Therefore, administration of HA, particularly LHA, may contribute to lung repair through these mechanisms.

Several clinical trials have previously investigated the use of HA for the treatment of various diseases, including chronic obstructive pulmonary disease, cystic fibrosis, and chronic rhinosinusitis. Although different HA molecular weights were applied, the routes of administration were primarily aerosol inhalation or nasal nebulization, which are both safe and convenient and have demonstrated clinical efficacy [19,41,42]. In the present study, the experimental design aimed to ensure that HA could reliably reach the lungs to exert therapeutic effects. Considering that rodents cannot be fitted with oro-nasal masks and cannot be forced to perform deep inhalation, intra-tracheal instillation was therefore selected as the most appropriate delivery method. For future clinical trials evaluating HA as a therapeutic strategy for acute respiratory distress syndrome (ARDS), aerosol inhalation would be the preferred route of administration.

## 4. Materials and Methods

### 4.1. Establishment of a Severe ARDS Animal Model

Male Sprague–Dawley (SD) rats (230–250 g) were anesthetized via the intraperitoneal injection of Zoletil 50 and Xylazine hydrochloride (Sigma, Cat. No. 23076359, Burlington, MA, USA). Following the confirmation of deep anesthesia, 5 mg of bleomycin (BLM; 1 unit activity/mg, Nippon Kayaku Co., Ltd., Tokyo, Japan) dissolved in 200 μL of sterile normal saline was instilled intratracheally into the left bronchus using a Hamilton syringe fitted with a polyethylene catheter (PE-10, I.D. 0.28 mm, O.D. 0.61 mm, length 2.5 cm). To ensure localized distribution, rats were maintained in a left lateral decubitus position tilted at 60° for 90 min. This procedure reliably established a severe and reproducible unilateral lung injury model consistent with ARDS.

### 4.2. Administration of Hyaluronan (HA)

Low-molecular-weight (MW) HA (LHA; sodium hyaluronate, MW ≅ 66–99 kDa, Lot#: 028842, Lifecore Biomedical, Chaska, MN, USA), medium-molecular-weight HA (MHA; sodium hyaluronate, MW ≅ 500–749 kDa, Lot#: 024051, Lifecore Biomedical, Chaska, MN, USA), and high-molecular-weight HA (HHA; Sodium Hyaluronate, MW ≅ 1.01–1.8 MDa, Lot#: 028605, Lifecore Biomedical, Chaska, MN, USA) were administered. Starting from Day 7 after BLM injection, HA was administered three times in Week 2 (Days 7, 9, and 11) and twice each in Weeks 3 (Days 14 and 17) and Week 4 (Days 21 and 24) (Figure 1A). For dosing, 200 μg of LHA, MHA, or HHA was dissolved in 200 μL of normal saline for administration; for the HA mixture (MIX HA), 67 μg of LHA, MHA, and HHA each were dissolved in 200 μL of normal saline. Dosing was performed via intratracheally injecting various types of HA into the anesthetized rats.

### 4.3. Experimental Groups

The animals were randomly assigned to one of the following six groups:

1. Normal group: On Day 0, normal rats were intratracheally administered 200 μL of saline. Starting from Day 7, only 200 μL of saline was intratracheally injected (Figure 1A).
2. BLM group: On Day 0, rats received an intratracheal injection of 5 mg of BLM. On Day 7 following BLM injection, only 200 μL of saline was intratracheally administered. No other treatment was given (Figure 1A).
3. BLM + LHA group: On Day 0, rats received an intratracheal injection of 5 mg of BLM. Starting from Day 7 after BLM injury, intratracheal injection of LHA was performed 7 times (Figure 1A).
4. BLM + MHA group: Rats received an intratracheal injection of 5 mg of BLM on Day 0. Starting from Day 7 after BLM injury, MHA was intratracheally injected 7 times (Figure 1A).
5. BLM + HHA group: Rats received an intratracheal injection of 5 mg of BLM on Day 0. Starting from Day 7 after BLM injury, HHA was intratracheally injected 7 times (Figure 1A).
6. BLM + MIX HA group: Rats received an intratracheal injection of 5 mg of BLM on Day 0. The HA mixture was intratracheally injected 7 times starting from Day 7 after BLM injury (Figure 1A).

For each group, weight, arterial SpO_2_, and respiratory rate were assessed weekly. The animals were sacrificed on Day 28 after BLM administration to observe the lung morphology and bronchoalveolar lavage cell counts. The animal groups and study flow chart are displayed in Figure 1A.

### 4.4. Assessments of Pulmonary Function

#### 4.4.1. Assessment of Arterial Oxygen Saturation (SpO_2_)

Rats were anesthetized using isoflurane (Baxter, Cat. No. 228–194, Deerfield, IL, USA). Arterial oxygen saturation (SpO_2_) was measured using a pulse oximeter (NONIN LS1-10R, Plymouth, MN, USA) with the sensor probe attached to the hind paw.

#### 4.4.2. Assessment of Partial Pressure of Arterial Oxygen (PaO_2_)/Carbon Dioxide (PaCO_2_)

Rats were anesthetized with isoflurane (Baxter 228-194, Deerfield, IL, USA), and arterial blood samples were collected into syringes preloaded with 30 IU heparin. Arterial partial pressures of oxygen (PaO_2_) and carbon dioxide (PaCO_2_) were then measured using a blood gas analyzer (Roche Cobas b 221, Basel, Switzerland)

#### 4.4.3. Assessment of Respiratory Rate

Rats were placed in a closed cylindrical detection chamber (Whole-Body Plethysmograph, emka Technologies, Paris, France), and the respiratory airflow was continuously recorded for 15 min using BIOPAC BSL 4.0 MP45 software. Resting respiratory rates were subsequently quantified from the recorded data.

### 4.5. Magnetic Resonance Imaging (MRI)

Rat lung images were acquired using a 7.0 T MRI scanner (BRUKER BIOSPEC 70/30, Billerica, MA, USA) at the Instrumentation Center, National Taiwan University. The thoracic cavity was scanned in the horizontal plane from rostral to caudal at 1.5 mm intervals until the entire cavity was covered, yielding 20–30 images per animal, depending on the initial scanning position. To minimize bias from variable slice numbers, the tracheal carina was designated as the anatomical reference point. For quantification, the slice containing the carina and the subsequent four slices were selected. The black area within the left thoracic cavity on these five slices was summed to represent the left lung area, and this value was then multiplied by the slice thickness (1.5 mm) to obtain the left lung volume for each rat.

### 4.6. Cell Counting of Broncho-Alveolar Lavage Fluid (BALF)

After phosphate-buffered saline (PBS) perfusion, the lungs and trachea of the rats were obtained. Polyethylene (PE) tubing (PE60, inner diameter: 0.76 mm, outer diameter: 1.22 mm) connected to a 20 G needle was inserted into the left lung via the left bronchus, and 0.5 mL of PBS was administered and aspirated. Subsequently, a second lavage was performed with another 0.5 mL of PBS, and 1 mL of BALF was obtained. The BALF was then centrifuged at 1500 rpm for 5 min. The supernatant was collected and stored at −20 °C for further analysis, whereas the cell pellet was resuspended in 1 mL of saline for cell counting.

### 4.7. Determination of Cytokine/Chemokine Concentrations

The concentrations of 26 cytokines were measured in rat sera using the MILLIPLEX^®^ RECYMAG65K27PMX Rat Cytokine/Chemokine Magnetic Bead Panel (EMD Millipore, Burlington, MA, USA). The panel included Eotaxin, Fractalkine, G-CSF, GM-CSF, interleukin (IL)-1α, IL-5, IL-17A, IL-18, interferon gamma-induced protein-10 (IP-10), Leptin, lipopolysaccharide-inducible CXC chemokine (LIX), MCP-1, MIP-1α, MIP-2, RANTES, TNFα, VEGF, EGF, IFNγ, IL-1β, IL-2, IL-4, IL-6, IL-10, IL-12p70, and IL-13. In brief, serum samples were incubated with antibody-conjugated beads on a plate shaker at room temperature for 2 h. After washing, detection antibodies were added and incubated for 1 h at room temperature, followed by incubation with streptavidin–phycoerythrin for 30 min. The supernatant was then discarded, the plates were washed, and the beads were resuspended in assay buffer. Cytokine levels were quantified using a Luminex^®^ 200TM analyzer (Austin, TX, USA).

### 4.8. Perfusion Fixation of Experimental Animals and Embedding Tissue into Paraffin Blocks

Rats were euthanized through intraperitoneal overdose of Zoletil 50 and Xylazine hydrochloride (Sigma, Cat. No. 23076359, Burlington, MA, USA). Following 4% paraformaldehyde perfusion, the left and right lungs were fixed at 4 °C for 2 days and subsequently processed for paraffin embedding. In brief, tissues were dehydrated through a graded ethanol series (70%, 80%, 95%, and 100% for 20 min each), cleared in xylene–paraffin mixtures (1:1 and 1:3), and infiltrated with pure paraffin prior to embedding.

### 4.9. Tissue Sectioning and Slice Collection

Lung tissues were serially sectioned sagittally from the outermost lateral side into 5 μm slices (see Supplemental Figure S1 for the collection methodology). Sections were floated on warm water (40–45 °C) to remove wrinkles, mounted onto microscope slides, and dried on a 50 °C hot plate.

### 4.10. Hematoxylin and Eosin (H&E) Staining

Lung tissue sections were deparaffinized before being stained with hematoxylin (Muto Pure Chemicals Co., Ltd., Cat. No. 3008-1, Tokyo, Japan) and eosin (Muto Pure Chemicals Co., Ltd., Cat. No. 3200-2, Tokyo, Japan). The left lung volume and alveolar airspace were quantified by summing all H&E-stained images (18 sections) and analyzing them using Image-Pro software (version 4.5.0.29, ipwin32; Media Cybernetics, Rockville, MD, USA). The percentage of cell infiltration was calculated as the mean value across all H&E-stained sections (Supplemental Figure S1, first panel).

### 4.11. Sirius Red Staining

After deparaffinization and rehydration, lung tissue sections were stained with 0.1% Sirius red (Sigma, Cat. No. 2610-10-8, St. Louis, MO, USA) in picric acid for 10 min (Supplemental Figure S1, second panel). Collagen deposition was quantified as the mean red-stained area ratio across all Sirius red-stained left lung sections using ImagePro software (version 4.5.0.29).

### 4.12. Protein Extraction

The entire left lung from each group was ground in a mortar in the presence of liquid nitrogen. Subsequently, 1 mL RIPA lysis buffer (Merck Millipore, Cat. No. 20-188, Burlington, MA, USA) with 10 μL Halt™ Protease & Phosphatase Inhibitor Cocktail (100X; Cat. No. 78440; Lot 3287120; Thermo Fisher Scientific Inc., Waltham, MA, USA) was added and reacted overnight until homogenized at 4 °C. The samples were centrifuged at 13,000 rpm at 4 °C for 30 min. The supernatant was obtained and maintained at −20 °C for storage.

### 4.13. Immunostaining and Western Blotting

Either a lung tissue slice or the nitrocellulose (NC) membrane was immersed in blocking solution (3% BSA and 5% normal goat serum in PBS) and reacted with primary antibodies diluted with blocking solution (anti-ED1 antibody [Merck Millipore, Cat. No. MAB1435, 1:400, Burlington, MA, USA], anti-CD86 antibody [Proteintech 13395-1-AP, 1:1000, Rosemont, IL, USA], anti-CD206 [Abcam ab64693, 1:1000, Cambridge, UK], anti-MMP9 [Abcam ab76003, 1:1000, Cambridge, UK], anti- MMP2 [Abcam ab92536, 1:1000, Cambridge, UK], anti-TLR4 [Abcam ab30667, 1:1000, Cambridge, UK]) at 4 °C for 12–18 h. Subsequently, the slice or the NC membrane was incubated with the corresponding secondary antibodies at room temperature for 1 h, followed by incubation with avidin-biotinylated-horseradish peroxidase complex (ABC kit, Vector Laboratories, Newark, CA, USA) at room temperature for 1 h. The slice or membrane was then washed with 0.01 M PBS three times, with 5 min per wash. Finally, DAB (5 mg DAB, 30% H_2_O_2_ 3.5 μL in 10 mL Tris-HCl pH7.4) was added for signal development.

### 4.14. Statistical Analysis

All experimental data are presented as the mean ± SEM (standard error of the mean). One-way analysis of variance (ANOVA) was applied to compare means among groups, followed by Tukey’s test for multiple comparisons. A *p*-value of less than 0.05 was considered to be statistically significant.

## 5. Conclusions

The combined administration of low-, medium-, and high-molecular-weight hyaluronan (LHA, MHA, and HHA), collectively referred to as MIX HA, may exert synergistic effects by integrating the distinct biological activities of each molecular form, thereby producing a superior therapeutic effect in ARDS. This strategy may provide an alternative clinical approach for the management of ARDS, having the potential to reduce disease-related mortality.

## Data Availability

The raw data supporting the conclusions of this article will be made available by the authors on request.

## Supplementary Materials

The following supporting information can be downloaded at: https://www.mdpi.com/article/10.3390/ijms27020580/s1.
