# Supplementary material for: Hyaluronan of Different Molecular Weights Exerts Distinct Therapeutic Effects on Bleomycin-Induced Acute Respiratory Distress Syndrome
Source: Int J Mol Sci. 2026 Jan 6;27(2):580. doi: 10.3390/ijms27020580 (PMC12840770; doi:10.3390/ijms27020580)

Supplemental Figure S1. Sectioning methodology of lung tissues and slice allocation for staining

The left lung was serially sectioned at a thickness of 5 µm. Sagittal sections were obtained starting from the outermost lateral surface of the lung. Ten consecutive sections were collected and placed sequentially on glass slides labeled A, B, C, D, E, F, G, H, I, and J, corresponding to sections No. 1 to 10, for different histological or immunohistochemical analyses. The next consecutive series (sections No. 31 to 40) were similarly mounted on slides A–J, and this process was repeated progressively toward the hilum of the lung until the entire left lung was sectioned. Thus, the horizontal row labeled A①–J① represents the outermost region of the left lung, whereas A⑥–J⑥ corresponds to regions near the pulmonary hilum. Sections in the A column (e.g., 1, 31, 61, 91, 121 – 871) were stained with hematoxylin and eosin (H&E) for general histological evaluation. Sections in the B column (e.g., 2, 32, 62, 92, 122 – 872) were subjected to collagen staining for assessing tissue fibrosis. Sections in the C column (e.g., 3, 33, 63, 93, 123 – 873) were immunostained with anti-ED1 antibody to evaluate inflammatory cell infiltration. Sections in the D column (e.g., 4, 34, 64, 94, 124 – 874) were immunostained with anti-α-SMA antibody to assess changes in myofibroblast distribution.

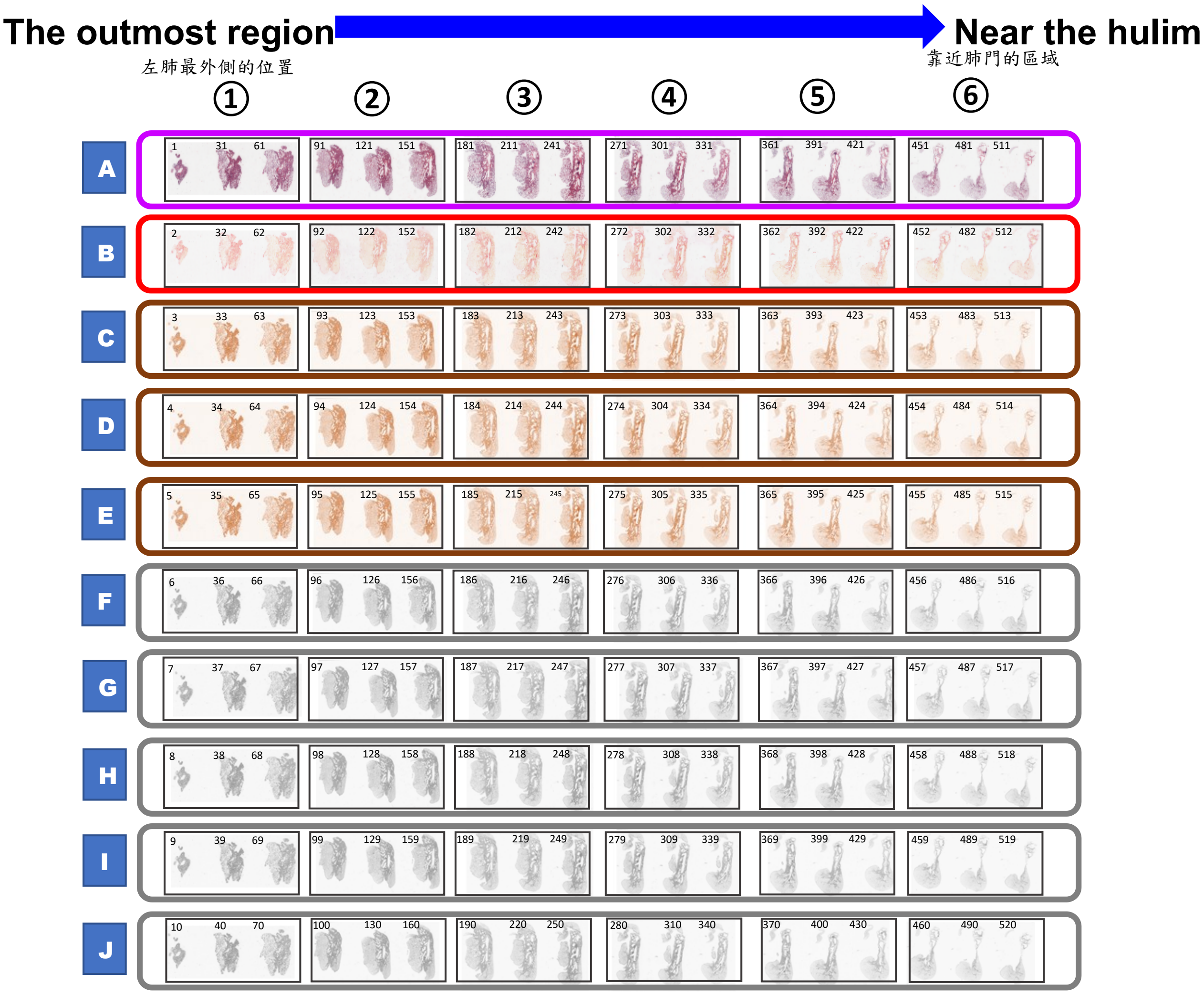

Supplement: Supplementary file 1 [file ijms-27-00580-s001.zip › ijms-4027374-supplementary.pdf]
